# Memory CD8 T Cells Protect against Cytomegalovirus Disease by Formation of Nodular Inflammatory Foci Preventing Intra-Tissue Virus Spread

**DOI:** 10.3390/v14061145

**Published:** 2022-05-25

**Authors:** Rafaela Holtappels, Jürgen Podlech, Kirsten Freitag, Niels A. Lemmermann, Matthias J. Reddehase

**Affiliations:** Institute for Virology and Research Center for Immunotherapy (FZI), University Medical Center of the Johannes Gutenberg University, 55131 Mainz, Germany; r.holtappels@uni-mainz.de (R.H.); podlech@uni-mainz.de (J.P.); kfreitag@uni-mainz.de (K.F.); lemmermann@uni-mainz.de (N.A.L.)

**Keywords:** adoptive cell transfer, antiviral protection, cytomegalovirus (CMV), growth kinetics, histopathology, immunotherapy, liver infection, memory CD8 T cells, nodular inflammatory focus (NIF), virus spread

## Abstract

Cytomegaloviruses (CMVs) are controlled by innate and adaptive immune responses in an immunocompetent host while causing multiple organ diseases in an immunocompromised host. A risk group of high clinical relevance comprises transiently immunocompromised recipients of hematopoietic cell transplantation (HCT) in the “window of risk” between eradicative therapy of hematopoietic malignancies and complete reconstitution of the immune system. Cellular immunotherapy by adoptive transfer of CMV-specific CD8 T cells is an option to prevent CMV disease by controlling a primary or reactivated infection. While experimental models have revealed a viral epitope-specific antiviral function of cognate CD8 T cells, the site at which control is exerted remained unidentified. The observation that remarkably few transferred cells protect all organs may indicate an early blockade of virus dissemination from a primary site of productive infection to various target organs. Alternatively, it could indicate clonal expansion of a few transferred CD8 T cells for preventing intra-tissue virus spread after successful initial organ colonization. Our data in the mouse model of murine CMV infection provide evidence in support of the second hypothesis. We show that transferred cells vigorously proliferate to prevent virus spread, and thus viral histopathology, by confining and eventually resolving tissue infection within nodular inflammatory foci.

## 1. Introduction

Human cytomegalovirus (hCMV) is the prototype member of the β-subfamily of the herpes virus family (for an overview, see [1]). Based on the strict host-species restriction of productive infection, hCMV cannot be studied experimentally in animal models [2,3,4]. This limits the investigation of hCMV pathogenesis and immune control to clinical studies in naturally infected immunocompetent volunteers and patients with CMV disease, whereas designed studies involving mutagenesis of the viral or host genomes are precluded by ethical concerns. Valuable information on specific aspects of hCMV pathogenesis has been provided by studies of hCMV natural strains and designed viral mutants in humanized mice with human tissue implants. Nonetheless, this model cannot fully reflect the entirety of functional human organs and a cytokine network that is not altered by xenogeneic differences [5,6,7] (for a discussion on humanized vs. conventional mouse models, see also [8]). As alternatives, for studying CMVs in their natural host, nonhuman primate models [9,10,11,12,13] and rodent models [14,15,16], of which the mouse model based on murine CMV (mCMV) is the most versatile in terms of manipulating host and virus genetics [8,17,18,19], proved helpful for identifying the basic principles of CMV biology and pathobiology. These principles are shared between different virus–host pairs, as the coevolution of CMVs with their respective hosts has led to adaptations with biological convergence (discussed in [1,8,20]).

In all CMV–host pairs, productive infection is rapidly controlled in the immunocompetent host by mechanisms of innate and adaptive immunity, followed by latent infection, referred to as “latency”. Latency is characterized by the absence of infectious virions but maintenance of nonreplicating, epigenetically silenced viral genomes with limited gene expression [21,22,23,24,25,26,27,28,29,30], from which reactivation to productive infection can occur when immune surveillance becomes compromised [31,32,33,34,35]. In contrast, in immunologically immature or in immunocompromised hosts, CMVs can spread unhindered between and within almost all organs, which leads to infection of many different cell types and hence to viral histopathology in many tissues, resulting in multiple organ failure [36,37,38]. All CMVs also have acquired an arsenal of “private genes” that are not homologous between CMV species. These genes are dedicated to subverting essentially all host defense mechanisms, including cell-intrinsic defense [39] as well as innate [40,41] and adaptive immunity [41,42,43,44,45,46]. Again, although the mechanisms of “immune evasion” differ markedly in molecular terms, there exist strong functional analogies.

The medical relevance of hCMVs results from birth defects after congenital infection of the immunologically immature fetus [47,48] as well as from infection under conditions of compromised immunity. Risk groups are immunocompromised transplantation patients. Recipients of allogeneic solid organ transplants (SOTs), who undergo immunosuppressive treatment for the prophylaxis or therapy of a host-versus-graft (HvG) response that can cause immune-mediated graft rejection, are at risk of graft failure from a reactivated latent hCMV infection. Notably, in SOTs, the reactivating virus is mostly donor-derived, which indicates that reactivation occurs more frequently in the transplanted organ from a latently infected donor and less frequently in the organs of a latently infected recipient [49,50,51,52]. An inverse risk profile is observed for recipients of hematopoietic cell transplantation (HCT), in whom hCMV reactivation occurs less frequently in hematopoietic cells of a latently infected donor and more frequently in organs of a latently infected recipient [52]. The source of reactivating virus in both SOT and HCT suggests the existence of a latently infected, nonhematopoietic tissue cell, with endothelial cell types being candidates (for a discussion of latently infected cell types, see [53]).

Clinical HCT is performed to reconstitute immune cells wiped out as collateral damage by hematoablative treatment, which actually aims at eradicating cells of aggressive hematopoietic malignancies that withstand standard antitumoral therapies. As a consequence, HCT patients are transiently immunocompromised until the immune system is fully reconstituted. As shown recently in the mouse model of experimental HCT and infection with mCMV [54], disparities between HCT donor and recipient in major (MHC/HLA) or minor (minor-HAg) histocompatibility antigens in allogeneic HCT (allo-HCT) prevent the reconstitution of high-avidity antiviral CD8 T cells (reviewed in [55]), which would be needed to overcome viral “immune evasion” (reviewed in [56]). In addition, reconstitution in clinical T cell-undepleted allo-HCT is impeded by immunosuppressive prophylaxis or therapy of graft-versus-host (GvH) disease. All these conditions create a “window of risk” that favors the reactivation of latent hCMVs [57] with clinical manifestations of CMV disease, of which interstitial pneumonia is the most frequent and often lethal [38,58,59,60]. This applies in particular to HCT patients infected with hCMV strains that have become refractory to established antiviral pharmacotherapy [61,62].

Initiated by preclinical studies in the mouse model ([63], reviewed in [64,65]), a preemptive cytoimmunotherapy by adoptive transfer of antiviral CD8 T cells (CD8-AT) has become the last resort to prevent lethal CMV disease in allo-HCT recipients infected with hCMV strains that are resistant to antiviral medication [66,67,68,69,70,71,72,73]. Both experimental [74,75,76] as well as clinical [77] CD8-AT revealed that remarkably few virus-specific memory CD8 T cells can control virus infection in almost all susceptible organs, provided that CD8-AT is performed as a so-called “pre-emptive therapy” shortly after experimental infection in the mouse model or shortly after virus reactivation detected by routine follow-up PCR-monitoring of hCMV DNA in clinical HCT.

Here we used the mouse model of CD8-AT to decide between two alternative explanations for the high antiviral efficacy of low numbers of transferred memory CD8 T cells: (i) an early elimination of relatively few productively infected cells at a local site before the released virus can disseminate to distant target issues, or (ii) clonal expansion to numbers sufficient for preventing intra-tissue viral spread after organs were colonized by the virus.

## 2. Materials and Methods

### 2.1. Mice, Virus, and the Route of Infection

Female BALB/cJ (haplotype *H-2^d^*) mice were bred and housed under specified-pathogen-free (SPF) conditions by the Translational Animal Research Center (TARC) at the University Medical Center of the Johannes Gutenberg-University Mainz. Immunocompetent CD8-AT donors and immunocompromised CD8-AT recipients were used at the age of 8 to 12 weeks. Intraplantar infection was performed by injection of 10^5^ plaque-forming units (PFU) of cell culture-propagated, purified mCMV (strain Smith, ATCC VR-1399, American Type Culture Collection, Manassas, VA, USA) into the left hind footpad.

### 2.2. Adoptive Transfer of Memory CD8 T Cells (CD8-AT)

CD8-AT recipients and no-AT control mice were 8-week-old female BALB/cJ mice, immunocompromised by hematoablative total-body γ-irradiation with a single dose of 6.5 Gy. Immunocompetent donors of CD8-AT were primed by intraplantar infection. Ten months later, CD8 T cells derived from the spleens of 15 donors, pooled to account for individual variance and sufficient cell yield, were purified by positive immunomagnetic cell sorting [78]. CD8-AT was performed by intravenous infusion of 1 *×* 10^5^ CD8 T cells at 4 hrs after the hematoablative conditioning, followed 2 hrs later by intraplantar infection.

### 2.3. Peptides and Quantitation of Functional Epitope-Specific Memory CD8 T Cells

Viral epitopes corresponding to antigenic peptides presented by MHC class-I (MHC-I) molecules K^d^, D^d^, and L^d^ are derived from the mCMV open reading frames m04, m18, M45, M83, M84, M105, m123/IE1, m145, and m164 (listed with their amino acid sequences and presenting MHC-I molecules in [64,65]). Custom peptide synthesis with a purity of >80% was performed by JPT Peptide Technologies (Berlin, Germany).

Immunomagnetically purified CD8 T cells derived from the spleens of CD8-AT donors served as responder cells in an IFNγ-based enzyme-linked immunospot (ELISpot) assay ([74], and references therein). In essence, for quantitating functional mCMV epitope-specific memory CD8 T cells, the corresponding synthetic peptides were exogenously loaded at the molar concentration of 10^−7^ M on P815 (*H-2^d^*) mastocytoma cells for serving as stimulator cells in the assay. Graded numbers of CD8 T cells were seeded with the peptide-loaded stimulator cells in triplicate microcultures. After 18 hrs of incubation, spots, each representing a specifically sensitized IFNγ-secreting cell, were counted using ImmunoSpot S4 Pro Analyzer (Cellular Technology Limited, Shaker Heights, OH, USA). Frequencies of responding cells and the corresponding 95% confidence intervals were determined by intercept-free linear regression analysis [74]. Calculations were performed with SPSS Statistics, Version 23.

### 2.4. Cytofluorometric Analyses

The enrichment of CD8 T cells by positive immunomagnetic cell sorting was verified by cytofluorometric quantitation of TCRβ^+^CD8^+^CD4^−^ lymphocytes. Unspecific staining was blocked with an unconjugated anti-FcγRII/III antibody (anti-CD16/CD32, clone 93; BioLegend, San Diego, CA, USA). For multicolor cytofluorometric analyses, specific staining was performed with PE-conjugated anti-TCRβ (clone H57-597; BD Biosciences, Franklin Lakes, NJ, USA), PE-Cy5-conjugated anti-CD8a (clone 53-6.7; eBiosciences, San Diego, CA, USA), and FITC-conjugated anti-CD4 (clone GK1.5; BioLegend). A lymphocyte live gate was routinely set in the forward vs. sideward scatter plot. Analyses were performed with flow cytometer FC500 and CXP analysis software (Beckman Coulter, Brea, CA, USA).

### 2.5. Quantitation of Tissue Infection and CD8 T Cell Infiltration

At indicated times after CD8-AT, infectious virus in spleen and lungs was quantitated in whole organ homogenates by a virus plaque assay performed on monolayers of mouse embryo fibroblasts under conditions of “centrifugal enhancement of infectivity” ([78], and references therein) to increase the sensitivity of detection.

To visualize and quantitate infection in the microanatomical context of host tissues, specifically in liver tissue, infected cells in tissue sections were identified by immunohistochemical (IHC) staining of the intranuclear viral immediate-early (IE) protein IE1 in red color [78]. For simultaneous detection and quantitation of tissue-infiltrating CD8 T cells, a two-color IHC (2C-IHC) [78] was performed by red staining of IE1 and black staining of the CD8 molecule using monoclonal antibody rat anti-mouse CD8a (clone 4SM15; eBiosciences). Total numbers of infected cells and tissue-infiltrating CD8 T cells were calculated from cells counted in representative tissue sections and extrapolated to the whole organ by using a mathematical formula that corrects for overestimation when the diameter *D* of the counted object is > the thickness *d* of the tissue slice (for a detailed explanation, see [79]).

### 2.6. In Situ Detection of Proliferating CD8 T Cells

For 2C-IHC of liver tissue sections (for staining protocols, see [78]), CD8 T cells were visualized by black staining of the CD8a molecule (see Section 2.5), and proliferating cells were identified by red staining of proliferating cell nuclear antigen (PCNA) using a species-cross-reactive mouse IgG2a(kappa) monoclonal antibody directed against human/mouse/rat PCNA (clone PC10; BD Biosciences).

### 2.7. Determination of Viral and Cellular Doubling Times and Number of Cell Divisions

Viral and cellular doubling times (vDT or cDT = log2/*a*) and the corresponding 95% confidence regions were calculated by linear regression analysis from the slopes *a* of log-linear growth curves [78,80,81] using GraphPad Prism version 6.04 for Windows, GraphPad Software.

The number of cell division was calculated as *n* = log_2_ [*N(t)/N(0)*], where *N(t)* = number of tissue-infiltrating CD8 T cells at time *t* after cell transfer, and *N(0*) = number of initially (*t* = 0) transferred viral epitope-specific memory CD8 T cells.

## 3. Results and Discussion

### 3.1. Experimental Protocol of CD8-AT and Specificity Composition of the Transferred Cells

**Figure 1:**
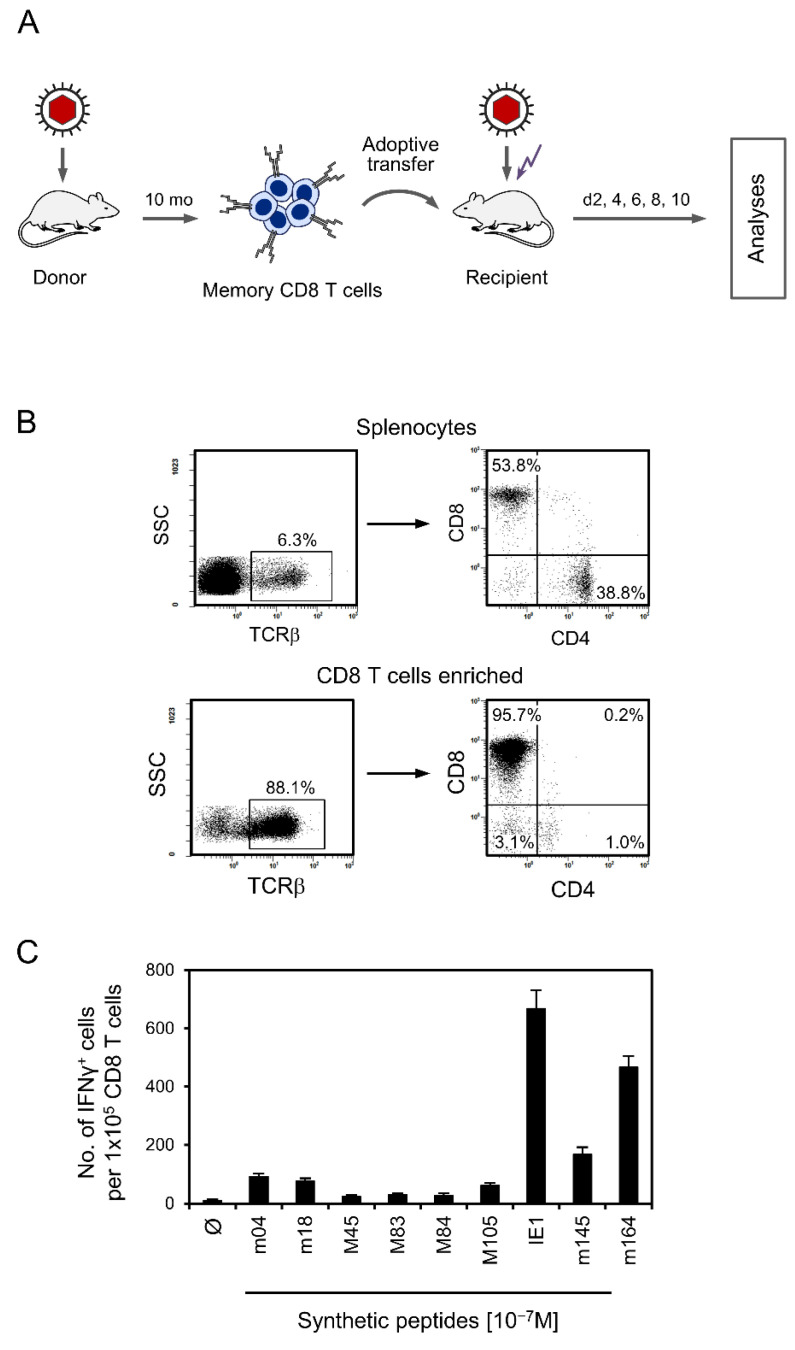
Characterization of the donor CD8 T-cell population used for CD8-AT. (**A**) Sketch of the experimental protocol. Polyclonal CD8 T cells were isolated from the spleen of donor BALB/c mice primed 10 months earlier by infection with mCMV. The cells were transferred into immunocompromised recipient BALB/c mice. (Flash symbol) γ-irradiation was applied to both recipients of CD8-AT and no-transfer control mice. (**B**) Cytofluorometric analysis documenting the enrichment of CD8 T cells by positive immunomagnetic cell sorting; (SSC) sideward scatter. (**C**) Frequencies of memory CD8 T cells specific for the viral epitopes indicated. Bars represent the frequencies of cells stimulated by viral antigenic peptides to secrete IFNγ in an ELISpot assay. Error bars represent the 95% confidence intervals.

As shown already in the pioneering work on CD8-AT in the mouse model [63], antiviral protection against acute infection is not mediated by naïve CD8 T cells within the timeline of AT but requires cells already primed and clonally expanded by antigen-specific sensitization. Subsequent studies using epitope deletion by replacement of the C-terminal MHC-I anchor residue of an antigenic peptide with alanine in the infecting virus [19] revealed a strict epitope-specificity of CD8 T-cell proliferation, tissue infiltration, and antiviral protection [75,76,79]. These findings thus excluded an antiviral bystander effector function of activated CD8 T cells with unrelated specificities. We, therefore, determined the number of mCMV epitope-specific CD8 T cells contained in the transferred CD8 T-cell population (Figure 1A–C).

As we have shown recently [82], local mCMV infection of immunocompetent mice does not induce the phenomenon of CD8 T-cell memory inflation (MI) that is observed after high-dose systemic infections, and that is characterized by a steady increase in KLRG1^+^CD62L^−^ inflationary T effector memory cells (iTEM) over time (reviewed in [83,84,85]). Instead, numbers of both iTEM and KLRG1^−^CD62L^−^ conventional T effector memory cells (cTEM) were found to decrease, whereas the population of T central memory cells (TCM) increased [82]. As all three subpopulations of specifically primed CD8 T cells have the potential to protect against mCMV infection, we have here used the total population and referred to it collectively as “memory” CD8 T cells. The panel of known antigenic mCMV peptides in the *H-2^d^* haplotype [64,65] almost covers the total memory response, as a viral genome-wide expression library [86] did not indicate the existence of unidentified antigenic peptides that would make a quantitatively significant contribution [87]. In accordance with the known immunodominance hierarchy of mCMV epitopes in the *H-2^d^* haplotype [87,88], the memory CD8 T-cell response was dominated by epitopes IE1 and m164 (Figure 1C). In total, ~1.6% of the CD8 T cells proved to be memory cells specific for known mCMV epitopes (Figure 1C) capable of becoming restimulated upon antigen re-encounter in the infected CD8-AT recipients.

### 3.2. The Time Course of Infection after CD8-AT Reveals Inhibition of Intra-Tissue Viral Spread

Protection against CMV histopathology and organ disease by CD8-AT performed with low numbers of virus-specific memory CD8 T cells is established experience from the experimental mouse model and clinical allo-HCT (see Section 1). It remained open to question whether CD8-AT operates primarily by preventing virus dissemination to the host tissues or by preventing virus spread within host tissues. These alternatives can be distinguished by an analysis of the protection kinetics.

As we have previously shown in a different context, namely the role of virion envelope glycoprotein complexes [80,81], a single-time determination of a difference in the magnitude of tissue infection compared with a control group with no interference cannot distinguish between inhibition of initial virus entry into tissue or inhibition of subsequent virus spread within the tissue. Both mechanisms end up in an absolute difference in viral load that increases over time. Only kinetics can unravel the target site at which antiviral control operates. Specifically, in immunocompromised mice, the virus replicates exponentially. This results in log-linear growth curves that allow the calculation of viral doubling times (vDT) from the slopes of the corresponding regression lines. Notably, vDT is a constant that differs between host organs but is independent of the measured parameter of infection, namely the amount of infectious virus, the copy number of viral genomes, or the number of infected cells in tissues [89]. Interference with virus dissemination to tissues leads to parallel-shifted regression lines with the same vDT, whereas interference with intra-tissue virus spread results in divergent regression lines and different vDT.

**Figure 2:**
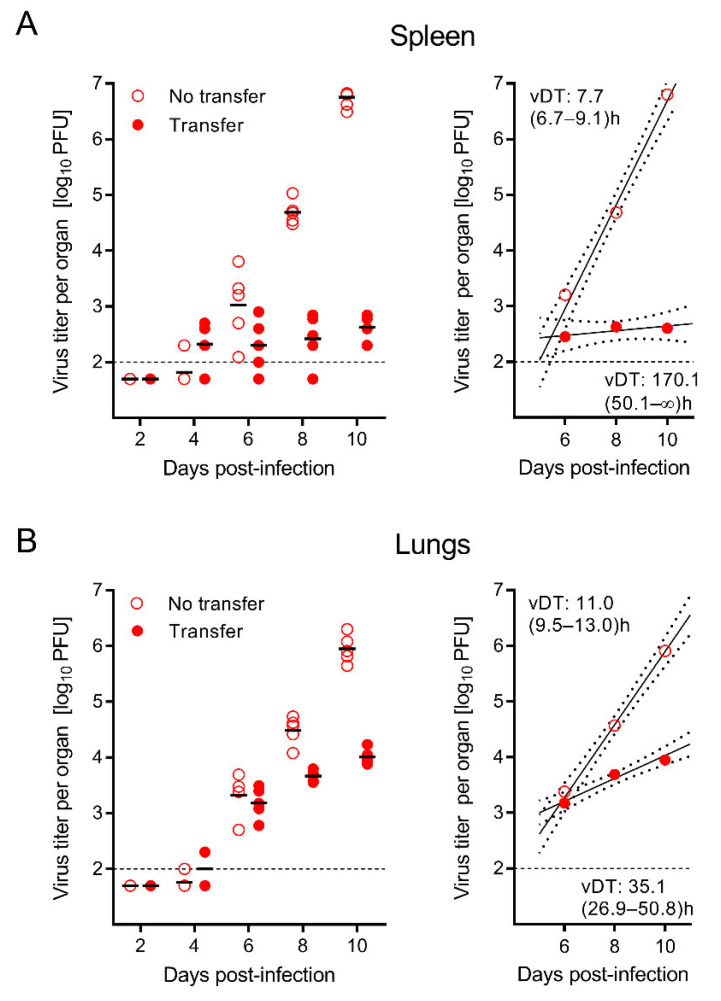
CD8-AT inhibits exponential virus growth reflected by a prolonged viral doubling time (vDT) of infectious virus in the spleen (**A**) and lungs (**B**). AT was performed with 10^5^ CD8 T cells, of which ~1.6% were memory cells specific for mCMV epitopes (recall Figure 1C). Symbols represent data from individual mice. Median values are marked. Red-rimmed empty circles—no AT; Red-filled circles—AT. Left panels—raw data. Right panels—calculated log-linear regression lines and the associated 95% confidence regions. The calculation was based on all data from individual mice at the indicated times. For clarity, symbols show only the median values. The dashed lines represent the detection limit of the virus plaque assay. vDT values represent the most probable values. The respective 95% confidence intervals for vDT are shown in parentheses.

Here we used this approach to test if CD8-AT, when compared to the no-AT group, leads to a parallel but down-shifted regression line with unaltered vDT or a divergent regression line with a reduced slope and thus slower vDT (Figure 2). The results for the infectious virus in the spleen (Figure 2A) and lungs (Figure 2B) clearly revealed a significantly higher vDT value, and thus a reduced infection, in the group with CD8-AT. This finding proved that virus-specific CD8 T cells prevented intra-tissue virus spread rather than viral dissemination to and entry into tissue.

**Figure 3:**
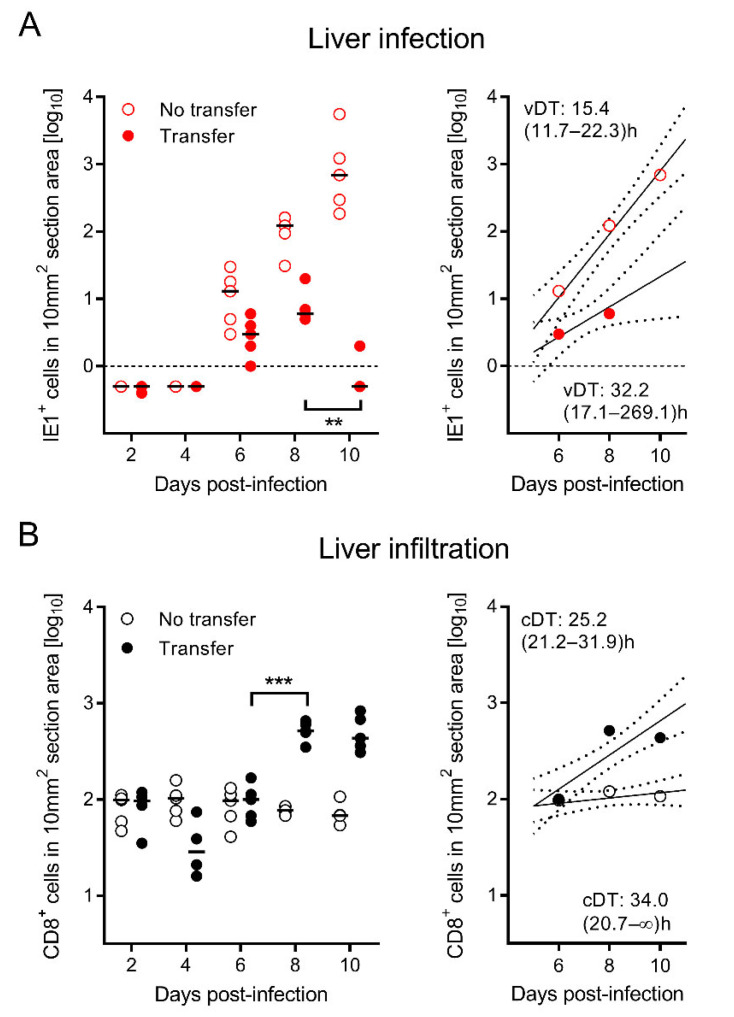
CD8-AT inhibits exponential virus spread in liver tissue reflected by a prolonged viral doubling time (vDT) of the number of infected liver cells, corresponding to enhanced tissue infiltration by CD8 T cells indicated by a shorter cellular doubling time (cDT). Data refer to the same experiment as shown in Figure 2. 2C-IHC was performed to quantitate infected liver cells (mostly hepatocytes) by red staining of the intranuclear viral protein IE1 (**A**) and tissue-infiltrating CD8 T cells by black staining of the CD8a molecule (**B**). Red- or black-rimmed empty circles—no AT. Red or black filled circles—AT. For further details, see the legend of Figure 2. The dashed lines in (**A**) represent the detection limit of the IE1-specific IHC. For comparisons of most interest, significance levels were determined. For the comparison of groups that include data below the detection limit, as is the case in (**A**), the distribution-free Wilcoxon–Mann–Whitney test was applied. Otherwise, the Student’s *t*-test with Welch’s correction of unequal variance was performed for log-transformed data in (**B**). Significant with *p* < 0.01 (**) or *p* < 0.001 (***).

In the same experiment, 2C-IHC was performed on liver tissue sections to simultaneously quantitate infected liver cells and liver-infiltrating CD8 T cells (Figure 3). The virus growth analysis was performed by quantitating infected liver tissue cells, which are mostly hepatocytes, identified by the expression of the intranuclear viral protein IE1 (Figure 3A). Again, regression lines were divergent with a slower vDT in the CD8-AT group, thus confirming the inhibition of intra-tissue virus spread. The corresponding infiltration of CD8 T cells was identified by the expression of the CD8 molecule (Figure 3B). Whereas liver-resident residual CD8 T cells in the no-AT control group stayed at a constant basal level throughout the time course, tissue infiltration by CD8 T cells derived from AT reached significance on day 8 and preceded the control of infection that became apparent on day 10 (Figure 3A,B). This inverse correlation indicates that liver-infiltrating AT-derived CD8 T cells limit virus spread even after liver infection has been established.

### 3.3. Tissue-Infiltrating CD8 T Cells Confine Infection to Nodular Inflammatory Foci (NIF)

**Figure 4:**
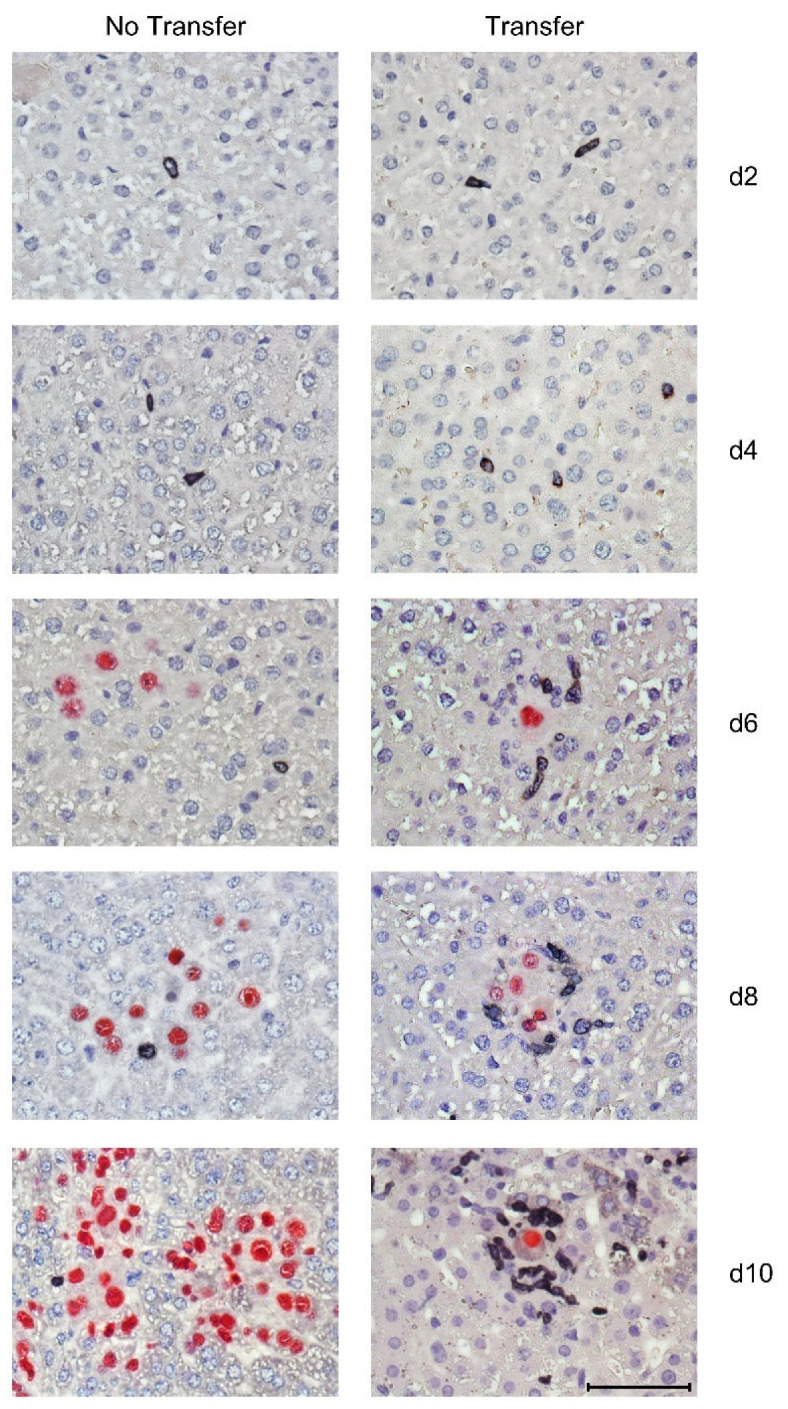
2C-IHC images showing the time course of NIF formation after CD8-AT. Infected liver cells, which are primarily hepatocytes, are identified by red staining of the intranuclear viral protein IE1. Tissue-infiltrating, NIF-forming CD8 T cells are identified by black staining of the CD8a molecule. Light hematoxylin counterstaining reveals the context of liver tissue. In the absence of CD8-AT, NIF are not formed, and the virus spreads unhindered. The bar marker represents 50 µm and applies to all images.

Tissue-infiltrating virus-specific CD8 T cells are not randomly distributed in tissue but aggregate at infected cells to form nodular inflammatory foci (NIF), confining the infection. This NIF formation, and thus the antiviral function, depends on the presentation of viral antigenic peptides by the surrounded infected cells [75,76,79] and requires high functional avidity of the CD8 T cells to overcome viral immune evasion mechanisms [56]. In the time course of the CD8-AT experiment for which liver tissue infection and CD8 T-cell infiltration were analyzed quantitatively (Figure 3), protective NIF formation in liver tissue coincided with the burst of CD8 T-cell infiltration between day 6 and day 8 after AT, whereas virus continued to spread unhindered, and thus led to disseminated tissue infection, when no AT was performed (Figure 4).

### 3.4. Most CD8 T Cells within NIF Express Proliferating Cell nuclear Antigen (PCNA)

The hypothesis that low numbers of transferred CD8 T cells must expand in the AT-recipients to control the virus intra-tissue spread in multiple host organs demands that AT-derived CD8 T cells proliferate. Previous work in the model of listeria infection has shown the “stemness” of central memory CD8 T cells with the capacity of an extensive expansion of low numbers of transferred cells for mediating protection [90,91]. As we have shown previously with epitope deletion mutants of mCMV [19], the presentation of viral antigenic peptides in AT recipients is essential for the expansion of low numbers of transferred viral epitope-specific CD8 T cells [79] and the formation of protective NIF [75,76,79]. It remained open to question if the cells proliferate only in lymphoid tissue before they infiltrate and form NIF in non-lymphoid tissue or if they also proliferate within NIF.

**Figure 5:**
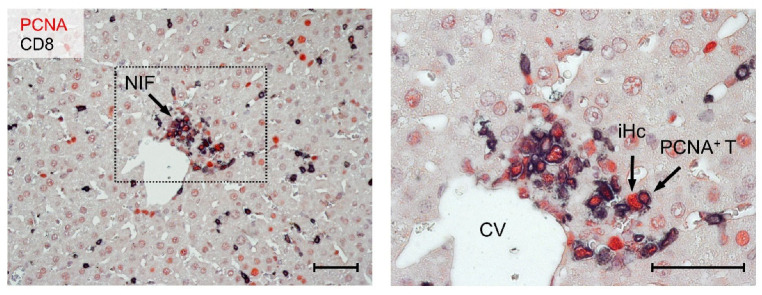
2C-IHC images showing proliferating CD8 T cells accumulated in a perivascular nodular inflammatory focus (NIF). Liver tissue-infiltrating CD8 T cells are identified by black staining of the CD8a molecule. Proliferating CD8 T cells are identified by red nuclear staining of PCNA and black cytoplasmic as well as membrane staining of CD8a. Infected liver cells, which are primarily hepatocytes, also express PCNA in their nuclei. (**Left image**) lower-magnification overview. (**Right image**) the framed area in the overview image is resolved to greater detail. Light hematoxylin counterstaining reveals the context of liver tissue. CV—central vein; iHc—infected hepatocyte; PCNA^+^ T—proliferating PCNA^+^ CD8 T cell. Bar markers, 50 µm.

Proliferating cell nuclear antigen (PCNA) is a reliable marker for proliferating cells, as it is expressed during the cell cycle in the G1 phase, reaching its maximum in the S phase and declining during the G2/M phase [92,93,94]. IHC specific for PCNA has the advantage of localizing proliferating cells in infected tissues, thus visualizing proliferating cells in their microanatomical context [95]. Notably, CMV infection is also known to induce PCNA [96]. We, therefore, used a 2C-IHC that distinguishes between infected hepatocytes expressing PCNA in their nuclei and proliferating CD8 T cells that coexpress intranuclear PCNA and the CD8 molecule that localizes to the cytoplasm and cell membrane (Figure 5).

In essence, on day 10 after infection and CD8-AT, most CD8 T cells within NIF expressed PCNA, indicating that they are not quiescent.

### 3.5. Control of Intra-Tissue Virus Spread Corresponds to Extensive CD8 T-Cell Expansion

**Figure 6:**
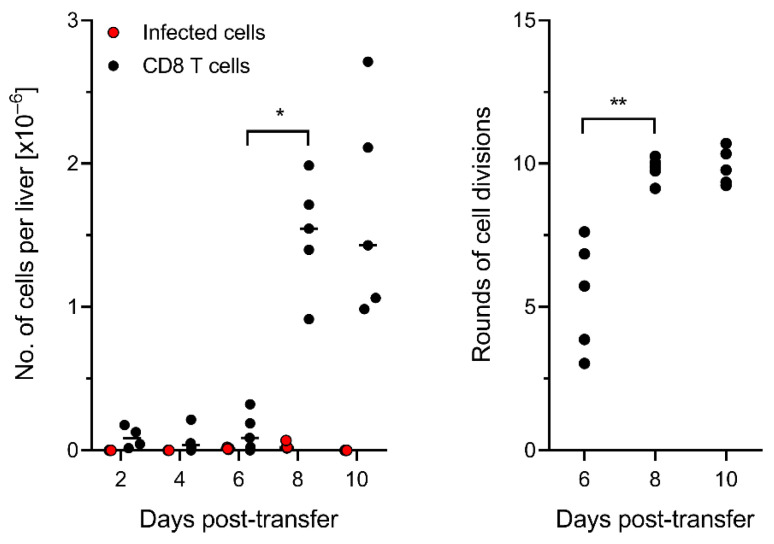
Extensive expansion of memory CD8 T cells in transfer recipients. (**Left panel**) for the indicated times after CD8-AT, infected IE1^+^ cells (filled red circles) and tissue-infiltrating CD8 T cells (filled black circles) were counted in representative 2C-IHC-stained liver tissue sections, and the absolute numbers of infected cells and CD8 T cells present in the whole liver were calculated by extrapolation. Symbols represent data of individual AT recipients. Median values are marked. (**Right panel**) calculated numbers of CD8 T-cell divisions based on initially transferred ~1600 viral epitope-specific cells. Symbols represent values for individual AT recipients. Median values are marked. As not all transferred cells home to the liver, the number of cell divisions represents a minimum estimate. Significant with *p* < 0.05 (*), calculated for log-transformed data. Significant with *p* < 0.01 (**), calculated for linear data. Student’s *t*-test with Welch’s correction of unequal variance.

The number of cell divisions that transferred cells undergo in the recipients cannot be estimated from the numbers of PCNA^+^ CD8 T cells present in tissues at a certain time, because a fraction of the cells that proliferated at some time in the period after AT might have lost the expression of PCNA and returned to quiescence at a later time. Thus, the best approach to determine T-cell expansion after AT is a quantitative input–output analysis of CD8 T cells, as described by us recently [79]. In essence, as only virus-epitope specific cells proliferate in response to antigen presentation after mCMV infection, we used the number of virus epitope-specific CD8 T cells contained in the transferred population (recall Figure 1C) as the input value and the numbers present in the whole liver at a certain time as the output value (Figure 6), expressed as absolute cell numbers (Figure 6, left panel) and as calculated rounds of cell division (Figure 6, right panel).

The data must be viewed as representing minimum estimates, as not all progeny of transferred CD8 T cells infiltrate the infected liver but infiltrate other infected organs as well. However, this has little influence on the overall conclusion, as every halving of the number of liver-infiltrating CD8 T cells would correspond to an underestimation by just one cell division.

In essence, the burst of liver infiltration took place between day 6 and day 8 after AT and corresponded, with variance, to about 10 rounds of cell division by day 8.

## 4. Conclusions

While many previous studies in the mCMV model have documented protection against viral histopathology by adoptively transferred antiviral CD8 T cells at a late stage, this is the first study that systematically relates control of tissue infection to tissue infiltration by antiviral CD8 T cells in the time course. The aim of this study was to explain how relatively low numbers of transferred cells can protect against infection of host organs. We considered two possible mechanisms. One idea was that few CD8 T cells may suffice to prevent virus dissemination to target tissues by eradicating the few initially infected cells, thus stopping productive infection at its very beginning. Alternatively, low numbers of transferred antiviral CD8 T cells might expand to high numbers, capable of fighting the infection even after multiple tissues have been colonized by the virus. This open question is now decided by the finding that adoptively transferred antiviral CD8 T cells expand extensively over time and operate primarily by inhibiting intra-tissue virus spread.

What can we learn from this new insight for clinical AT in HCT recipients in which hCMV reactivates? AT is performed as a so-called “pre-emptive” immunotherapy as soon as follow-up monitoring by quantitative PCR detects virus reactivation. Although this strategy should remain the first choice, our data indicate that AT therapy is promising even when the reactivated virus has already disseminated to the end-point target organs of CMV disease.

## Data Availability

The data presented in this study are available on request from the corresponding author.
